# Visual Crowding Reveals Field- and Axis-Specific Cortical Miswiring After Long-Term Axial Misalignment in Strabismic Patients Without Amblyopia

**DOI:** 10.1167/iovs.64.1.10

**Published:** 2023-01-18

**Authors:** Yiru Huang, Zitian Liu, Zidong Chen, Zongyi Zhan, Le Gao, Jingyi Hu, Yanyan Wu, Fang-Fang Yan, Daming Deng, Chang-Bing Huang, Minbin Yu

**Affiliations:** 1 State Key Laboratory of Ophthalmology, Zhongshan Ophthalmic Center, Sun Yat-sen University, Guangdong Provincial Key Laboratory of Ophthalmology and Visual Science, Guangdong Provincial Clinical Research Center for Ocular Diseases, Guangzhou, Guangdong, China; 2 Key Laboratory of Behavioral Science, Institute of Psychology, Chinese Academy of Sciences (CAS), Beijing, China; 3 Department of Psychology, University of Chinese Academy of Sciences, Beijing, China; 4 Shenzhen Eye Hospital, Shenzhen Eye Institute, Shenzhen Eye Hospital affiliated to Jinan University, Shenzhen, China; 5 School of Optometry, Shenzhen University, Shenzhen, China

**Keywords:** visual crowding, strabismus, long-term axial misalignment, cortical miswiring

## Abstract

**Purpose:**

Inspired by physiological and neuroimaging findings that revealed squint-induced modification of cortical volume and visual receptive field in early visual areas, we hypothesized that strabismic eyes without amblyopia manifest an increase in critical spacing of visual crowding, an essential bottleneck on object recognition and reliable psychophysical index of cortical organization.

**Methods:**

We used real-time eye tracking to ensure gaze-contingent display and examined visual crowding in patients with horizontal concomitant strabismus (both esotropia and exotropia) but without amblyopia and age-matched normal controls.

**Results:**

Nineteen patients with exotropia (12 men, mean ± SD = 22.89 ± 7.82 years), 21 patients with esotropia (10 men, mean ± SD = 23.48 ± 6.95 years), and 14 age-matched normal controls (7 men, mean ± SD = 23.07 ± 1.07 years) participated in this study. We found that patients with strabismus without amblyopia showed significantly larger critical spacing with nasotemporal asymmetry in only the radial axis that related to the strabismus pattern, with exotropia exhibiting stronger temporal hemifield crowding and esotropia exhibiting stronger nasal hemifield crowding, in both the deviated and fixating eyes. Moreover, the magnitude of crowding change was related to the duration and degree of strabismic deviation.

**Conclusions:**

Using visual crowding as a psychophysical index of cortical organization, our study demonstrated significantly greater peripheral visual crowding with nasotemporal asymmetry in only the radial axis in patients with strabismus without amblyopia, indicating the existence of hemifield- and axis-specific miswiring of cortical processing in object recognition induced by long-term adaptation to ocular misalignment.

For binocular creatures, the veridical representation of and effective interaction with the outside world depend heavily on the correspondence of both foveal and peripheral inputs from their two eyes. If the visual axes of the two eyes are misaligned, binocular activation of cortical neurons (at least in V1) is disrupted, with the fixating eye neutralizing the neural response from the deviated eye through interocular suppression.^1^^,^^2^ Without timely and appropriate treatment, strabismus can develop, a disorder affecting 1.93% of the population.^3^

Strabismus appearing early in life may lead to abnormal binocular functions, such as defective stereopsis, interocular suppression, and abnormal retinal correspondence, most of which are related to binocular coordination and/or oculomotor functions.^4^^–^^6^ Monocular functions in the deviated eye (e.g. visual acuity and contrast sensitivity), on the other hand, are usually spared.^7^ Here, inspired by physiological and neuroimaging findings of squint-induced modification of cortical volume and visual receptive field in early visual areas,^8^^,^^9^ we utilized a gaze-contingent paradigm to show that visual crowding, an essential bottleneck of object recognition and reliable psychophysical index of cortical organization,^10^^–^^12^ is asymmetrically impaired in the nasal and temporal visual fields of both the fixating and deviated eyes in older children and adults with strabismus but without amblyopia, showing specific cortical miswiring that follows long-term axial misalignment.

Visual crowding, the identification inefficiency for objects in clutter (Figs. 1A, 1B), affects the recognition of natural scenes and sets a fundamental limit on conscious visual perception.^11^^,^^13^^,^^14^ The magnitude of the crowding effects, usually indexed by critical spacing (i.e. the minimum distance between the center of the target and the center of the flankers required for correct discrimination), is proportional to the eccentricity of the target object and exhibits a signature radial–tangential anisotropy (Figs. 1C–E).^15^^–^^17^ Early work has demonstrated crowding as the neuroanatomic limits of cortical processing of object recognition,^11^^,^^18^ although the exact neural sites responsible may extend from V1 to downstream areas,^10^^,^^11^ and the extent of critical spacing is correlated with the cortical magnification factor.^19^ On the other hand, patients with strabismus exhibit atrophy of white and gray matter volume in the brain,^20^ and disrupted brain functional connectivity.^21^ We thus hypothesized that patients with strabismus without amblyopia would manifest an increase in critical spacing of visual crowding despite exhibiting normal visual acuity.

**Figure 1.**
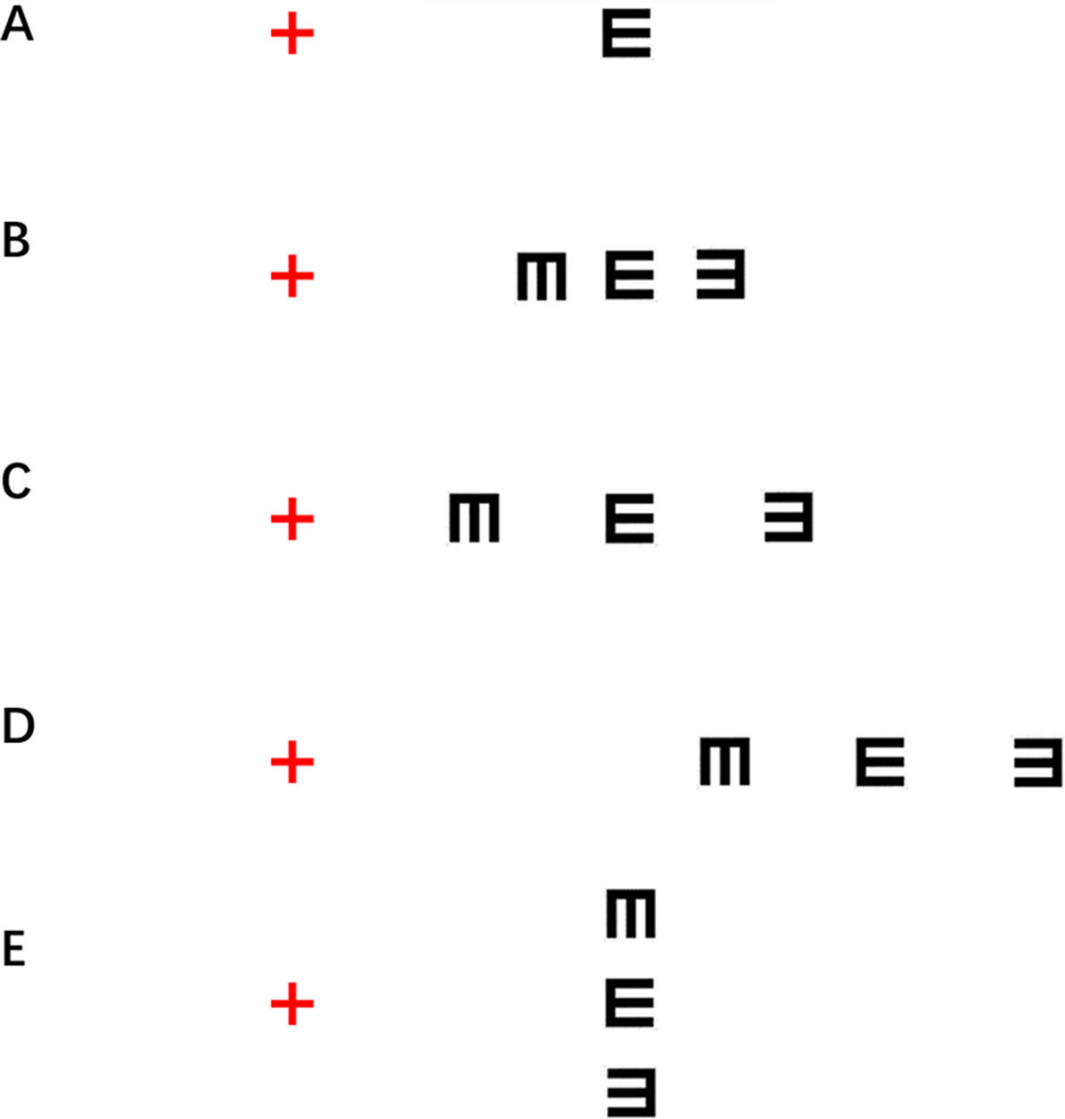
Characteristics of crowding in the peripheral vision. (**A**) Object recognition in isolation. When fixating on the red cross in an appropriate viewing distance, it is easy to identify the orientation of the tumbling E. (**B**) Crowding effects. It becomes harder to discriminate the orientation of the central target tumbling E when flanked (crowded) by other letters. (**C**) Flanker-target distance (center to center) dependence. When the two flankers are separated from the central target tumbling E by a larger distance, the orientation of the central E can be perceived clearly. (**D**) Eccentricity dependence. When the central target tumbling E becomes more eccentric, it becomes much harder to perceive even with the same flanker-target distance. (**E**) Radial–tangential anisotropy. For the same separation between the center of the flankers and the center of the target as in **B**, it is easier to identify the orientation of the tumbling E when the two flankers are tangential to fixation than when the two flankers are radial to fixation.

In the present study, we used real-time eye tracking to ensure a gaze-contingent display of the target and flankers at four different visual field locations and compared the critical spacing of patients with horizontal concomitant strabismus (both esotropia and exotropia) but without amblyopia with that of age-matched normal controls. Interestingly, we found that patients with strabismus with normal visual acuity showed significantly larger critical spacing with nasotemporal asymmetry in only the radial axis and the deviating direction, with exotropia exhibiting stronger temporal hemifield crowding and esotropia exhibiting stronger nasal hemifield crowding, in both the deviated and fixating eyes. This field- and axis-specific pattern of differences in crowding was related to the duration and degree of axial misalignment.

## Methods

This research followed the guidelines of the Declaration of Helsinki and was approved by the Institutional Review Board of Zhongshan Ophthalmic Center, Sun Yat-sen University. Informed consent was obtained from participants or their parents/legal guardians after explaining the purposes, procedures, risks, and benefits of this study.

### Participants

Nineteen patients with exotropia (12 men, mean ± SD = 22.89 ± 7.82 years), 21 patients with esotropia (10 men, mean ± SD = 23.48 ± 6.95 years), and 14 age-matched normal controls (7 men, mean ± SD = 23.07 ± 1.07 years) participated in this study. Patients were recruited through the Zhongshan Ophthalmic Center, Guangzhou, China. All participants underwent a comprehensive eye examination on a visit before the psychophysical tests, including determination of the best-corrected distance visual acuity, cycloplegic refraction to determine the best refractive correction, slit-lamp and funduscopic examinations, alternate prism cover tests at near and distance fixations, examination of eye movements, the Bagolini striated lens test, synoptophore evaluation, and the stereo acuity test (Vision Assessment Corporation Co., Elk Grove Village, IL, USA) to assess the binocular vision. Patients were diagnosed with horizontal concomitant strabismus by ophthalmologists at Zhongshan Ophthalmic Center. Horizontal concomitant strabismus is defined as a horizontal deviation of the visual axis and an identical angle of deviation of the squinting eye relative to the other eye, regardless of the direction of the gaze.^22^^–^^24^ An alternate prism cover test was performed at near and distance fixations to ensure that the ocular deviation was along only the horizontal direction and present at both near and distance. All patients had alternating strabismus with shifted fixation between their two eyes. Patients with clinically significant refractive error were instructed to wear spectacles for at least 3 months according to the Preferred Practice Patterns of the American Academy of Ophthalmology^25^ before the experiment. Only patients who had normal or corrected-to-normal visual acuity (0.00 logMAR or better) after refractive correction were included in this study. All patients reported having a history of strabismus since early childhood (4.20 ± 2.22 years) and had never undergone strabismus surgery. None of the patients had vertical, paralytic or restrictive strabismus, accommodative esotropia, acute concomitant esotropia, nystagmus, or a history of ocular surgery or prism correction. No patients had any neurological disorders or systemic diseases. Clinical characteristics of the participants are summarized in the Table. Clinical characteristics of each patient are listed in the Supplementary Table S1.

**Table.** Summarized Clinical Details of Different Groups

Clinical Details	Normal Controls	Exotropia Group	Esotropia Group
Number	14	19	21
Male sex, *n* (%)	7 (50.0)	12 (63.2)	10 (47.6)
Age (y)			
Range	20–26	12–37	13–33
Mean ± SD	23.07 ± 1.07	22.89 ± 7.82	23.48 ± 6.95
Spherical equivalent (diopters), mean ± SD			
Nondominant/deviated eye	−2.20 ± 2.17	−0.76 ± 2.13	1.18 ± 4.16
Fixating eye	−2.10 ± 2.04	−0.67 ± 1.60	0.76 ± 3.24
Best-corrected visual acuity, logMAR			
Nondominant/deviated eye	−0.01 ± 0.04	−0.01 ± 0.04	−0.01 ± 0.03
Fixating eye	−0.01 ± 0.03	−0.01 ± 0.04	−0.01 ± 0.03
Degree of strabismus (prism diopters)			
Degree range	–	XT 30–80	ET 20–80
Mean ± SD	–	52.00 ± 14.03	53.80 ± 20.17
Age of onset of strabismus			
Range	–	0–10	0–11
Mean ± SD	–	3.42 ± 3.44	3.05 ± 3.26
Duration of strabismus			
Range	–	9–30	9–31
Mean ± SD	–	18.63 ± 7.59	19.75 ± 8.76
Measurable stereoacuity, *n* (%)	14 (100)	3 (15.8)	0 (0)
Stereoacuity, arcsecs			
Arcsec range	20–40	40–200	nil
Mean ± SD	21.5 ± 2.42	113.33 ± 80.83	nil
Retinal correspondence test			
Synoptophore test: no. of NRC, *n* (%)	14 (100)	3 (15.8)	3 (14.3)
Bagolini test: no. of NRC, *n* (%)	14 (100)	7 (36.8)	8 (38.1)

XT, exotropia; ET, esotropia; NRC, normal retinal correspondence.

### Apparatus and Stimuli

Experimental programs were executed on a personal computer running MATLAB (MathWorks, Inc., Natick, MA, USA) and Psychtoolbox (version 3.0.14).^26^ Test stimuli were presented on a gamma-corrected liquid crystal display screen with a resolution of 1920 × 1080 and a refresh rate of 144 Hz. Participants seated at an eye-to-screen distance of 57 cm. A chin-and-forehead rest was used to limit head movement.

**Figure 2.**
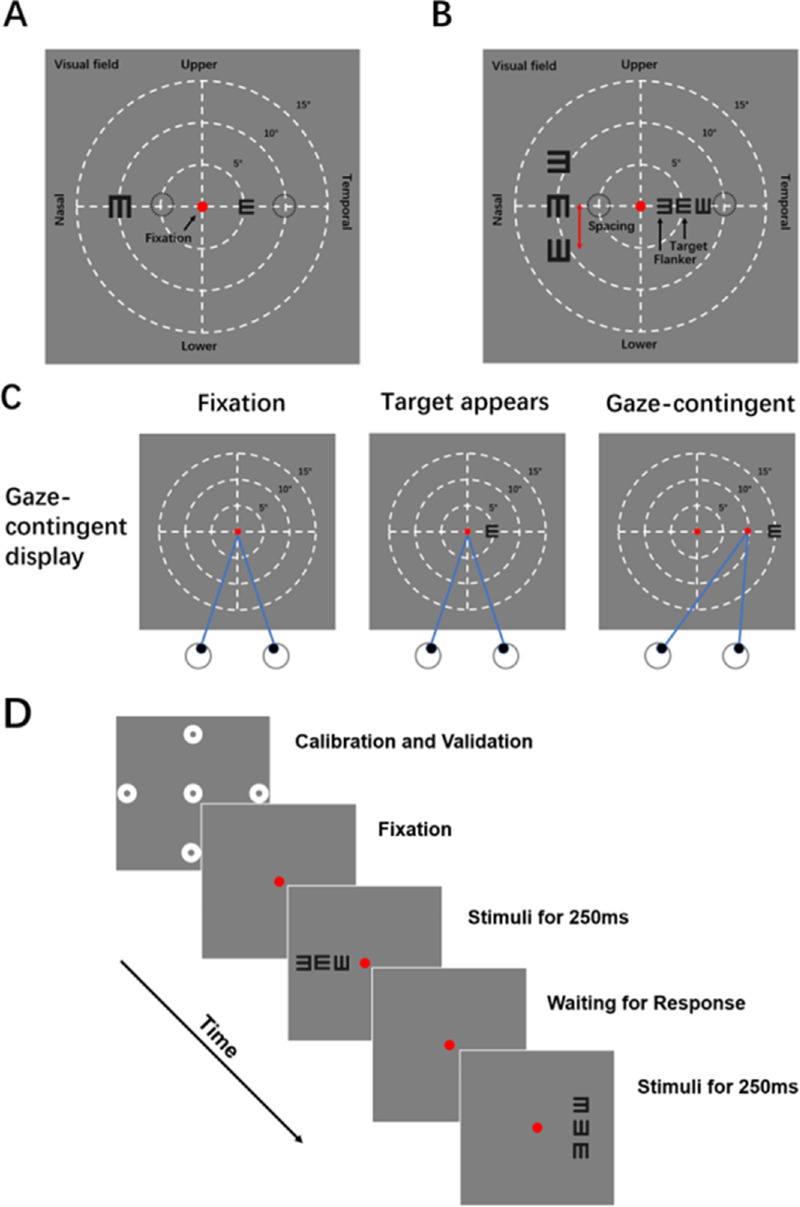
(**A**) Illustration of the stimuli used in the visual acuity test. Visual acuity was measured in the nasal and temporal hemifields at two eccentricities (5° and 10°), respectively. (**B**) Illustration of the stimuli used in the crowding test. The stimuli were consisted of a target tumbling E flanked by two tumbling Es appearing on both sides of the target along the radial or tangential axis. The critical spacing was measured at the same locations as the visual acuity test. (**C**) Gaze-contingent display. The participant gazed at the red dot in the center of the screen, waiting for the target letter appearing and then responding. A gaze-contingent paradigm was used to ensure display of the target and flankers in the specific visual field locations. Once the eye tracker detected participant's involuntary eye movement to the target letter, the program will move the target and flanker letters at the identical direction and distance to offset the involuntary eye movement. (**D**) Time course of the test. After a standard five-point calibration and validation, trials began with a central red fixation point presenting throughout the test. The stimuli appeared for 250 ms and randomly interleaved by different visual field locations, followed by an interstimulus interval for participants to respond without time limitation. After the response, there was a brief pause of 1 second and then the next trial started.

The stimuli consisted of a single black tumbling E in the visual acuity test (Fig. 2A) and a trigram of black tumbling Es in the crowding test (Fig. 2B), displayed on a uniform gray background (54 cd/m^2^). In the crowding test, the middle tumbling E was designated as the target and participants were asked to report its orientation (left, right, up, or down) by pressing the appropriate key on the keyboard. The trigrams were arranged along two different axes (radial and tangential) in the nasal or temporal hemifield, resulting in four testing conditions: (1) radial flankers in the nasal hemifield, (2) tangential flankers in the nasal hemifield, (3) radial flankers in the temporal hemifield, and (4) tangential flankers in the temporal hemifield. These four testing conditions appeared randomly at the eccentricity of 5° or 10°.

### Eye Movement Recording

A high-resolution infrared-emitting video-based eye tracker with a sampling rate of 1000 Hz (EyeLink 1000; SR Research, Ottawa, Canada) and a maximum spatial resolution of 0.02° was used to continuously monitor participants’ horizontal and vertical eye positions. A standard 5-point calibration and validation sequence was performed and repeated at the beginning of every test until the validation error was smaller than 1° on average.^27^ The average gaze error was 0.5°, ranging from 0.1° to 1°. A gaze-contingent paradigm was used to ensure that the target and flankers were displayed in the specific visual field locations (Fig. 2C). Once the eye tracker detected the participant's involuntary eye movement to the target letter, the program moved the target and flanker letters in the same direction and at the same distance to offset the involuntary eye movement. A chin-and-forehead rest was used throughout the experiment to minimize head movements and trial-to-trial variability in the estimate of gaze position. A good fixation was defined as at least 250 ms of stable gaze and position not exceeding 0.5° in any direction.^27^ Trials that failed to meet the requirements of good fixation were repeated until the requirements were met.

### Design

The test was monocular with the initial eye randomly selected and the untested eye covered by a black patch. To ensure that participants could see the target clearly in isolation, a visual acuity test with single tumbling E was first conducted to obtain the minimum letter size visible to the participants in a three-down-one-up staircase procedure.^28^ Next, we performed the crowding test, in which the initial letter size was set to 1.5 times the minimum letter size estimated from the visual acuity test and the initial spacing between the center of the target and the center of the flankers was 0.75 times the eccentricity.^29^ We first performed the crowding test with an eccentricity of 5° to obtain the critical spacing of crowding along radial and tangential axes in the nasal and temporal visual fields and then repeated the crowding test at an eccentricity of 10° in the same manner. After these tests, the tested eye was switched and the above process was repeated, leading to a total of sixteen crowding measurements (2 axes × 2 visual fields × 2 eccentricities × 2 eyes).

### Procedure

Participants first underwent a standard five-point calibration and validation procedure^30^ to provide a measurement of positions for the tested eye. During the test, participants were instructed to fixate on a central red point. Stimuli were briefly presented for 250 ms, and 4 different test conditions were randomly interleaved. Participants were asked to indicate the orientation of the target tumbling E by pressing the corresponding key on a computer keyboard. There was a brief pause of 1 second after each response, and then the next trial started (see Figs. 2C, 2D). A three-down one-up staircase procedure with 10% step size^31^ was adopted to track the spacing between the center of the target and the center of the flankers; specifically, 3 consecutive correct identifications of the orientation of the target tumbling E resulted in a decrease in the center-to-center distance between the target and flankers by 10% (i.e. *Distance*_t+1_ = *Distance*_t_ × 90%), whereas one incorrect identification resulted in an increase in the spacing by 10% (i.e. *Distance*_t+1_ = *Distance*_t_ × 110%). The staircase procedure was ended after six reversals and the mean separation of the last four reversals was taken as the critical spacing value (i.e. the space between the center of the target and the center of the flankers required for 79.4% correct identification).

Prior to the test, it was well explained, and the participants were given 10 minutes to practice. Each test in a specific visual field location took approximately 6 minutes. Between tests, the participants could elect to take a short break to avoid fatigue. Participants who could not complete the test well or could not maintain good fixation stability during the test were excluded. Ocular dominance was determined with Mile's test.^32^^,^^33^ For patients with strabismus, dominant eye means the fixating eye (FE) and nondominant eyes means the deviated eye (DE) which tends to turn more frequently. Due to personal reasons, some patients did not have enough time to complete the tests of both eyes. Thus, a total of 14 normal controls completed tests for both eyes; 19 patients with exotropia (19 out of 19) completed the test with their DE, and 13 (out of 19) patients with exotropia completed the test with their FE; and 21 patients with esotropia completed the test with their DE, and 16 (out of 21) patients with esotropia completed the test with their FE.

### Statistical Analysis

To reduce the influence of eccentricity,^34^ we first normalized the critical spacing by dividing the values by the eccentricity at which they were collected to obtain the normalized critical spacing (NCS). An analysis of variance (ANOVA), with axis (radial or tangential), eccentricity (5° or 10°), visual field (nasal or temporal) and eye (dominant/nondominant eye for normal controls, fixating/deviated eye for exotropia and esotropia patients) as within-subject factors and group (normal control, and exotropia, or esotropia) as between-subject factors, was performed for participants with data available on both eyes (i.e. 14 normal controls, 13 patients with exotropia, and 16 patients with esotropia) to examine whether the normalized critical spacing was differed according to axis, eccentricity, hemifield, and eye in each group.

Because we found that the NCS was comparable in both eyes and both eccentricities across groups (see Supplementary Table S2), the NCS was collapsed across eyes and eccentricities. First, the data measured at the corresponding visual field locations were combined across different eyes. For example, the NCS in the nasal visual field (i.e. temporal retina) at an eccentricity of 5° of the deviated eye was combined with the NCS in the nasal visual field (i.e. temporal retina) at an eccentricity of 5° of the fixating eye to obtain the mean NCS; a similar calculation was performed for the NCS at an eccentricity of 10° of 2 eyes. For subjects with monocular data, we retained monocular data for all relevant analyses. Then, the mean NCS at the same visual field location was combined across two eccentricities (5° and 10°) to obtain the final NCS for the following analysis with an analysis of variance to determine the crowding effects according to group, axis, and visual field. To confirm that retaining subjects with only monocular data did not influence our main results, we performed the analysis with data from only subjects with binocular data and this analysis produced similar results (see Supplementary Table S4).

Horizontal concomitant strabismus turns the visual axis of the eye toward either the nasal (e.g. esotropia) or temporal (e.g. exotropia) visual fields. To better characterize the visual crowding across different visual fields, we calculated the ratio of the NCS between the nasal and temporal hemifields (or the critical spacing ratio [CSR]) as follows: CSR = (NCS_nas_ − NCS_temp_)/(NCS_nas_ + NCS_temp_). CSR values of <0, 0, and >0 indicate greater crowding effects in the temporal field, symmetrical crowding effects in both visual fields, and greater crowding effects in the nasal field, respectively.^35^ An ANOVA was performed to determine if the CSR differed significantly according to axis or group, and a one sample *t*-test was used to examine whether the CSR was different from zero. A two-tailed Pearson correlation analysis was performed to examine the potential relationships among the duration of strabismus, degree of ocular deviation and the CSR.

The typical visual crowding exhibits a signature radial–tangential anisotropy.^12^ Therefore, we also calculated the radial-tangential anisotropy index (e.g. (NCS_rad_ - NCS_tan_)/(NCS_rad_ + NCS_tan_)). An ANOVA was performed to test if the radial-tangential anisotropy index differed significantly across hemifields and groups. A two-tailed Pearson correlation analysis was performed to examine the potential relationships among the duration of strabismus, degree of ocular deviation, and the anisotropy index.

All statistical analyses were performed using IBM SPSS version 25 (IBM, Armonk, NY, USA) and JASP 0.14.1.0 (https://jasp-stats.org), with *P* < 0.05 as the threshold for statistical significance.

## Results

An analysis of variance revealed that the normalized critical spacing varied significantly with hemifield (F_1,40_ = 5.73, *P* = 0.02), axis (F_1,40_ = 629.26, *P* < 0.001), and group (F_2,40_ = 9.33, *P* < 0.001) but not eye (F_1,40_ = 0.001, *P* = 0.98) or eccentricity (F_1,40_ = 1.25, *P* = 0.27; see Supplementary Table S2, Figure S1 for details). Because the interaction of eye and group (F_2,40_ = 1.14, *P* = 0.33), and eccentricity and group (F_2,40_ = 1.13, *P* = 0.33) were nonsignificant, our results indicate that the normalized critical spacing was comparable across the two eyes and the two eccentricities in healthy subjects, and subjects with esotropia and exotropia. The mean normalized critical spacing across eyes and eccentricities (i.e. averaged the data of the same hemifield and axis from the two eccentricities and eyes) was thus used in the following analysis.

### Stronger Visual Crowding is Both Hemifield- and Axis-Specific

**Figure 3.**
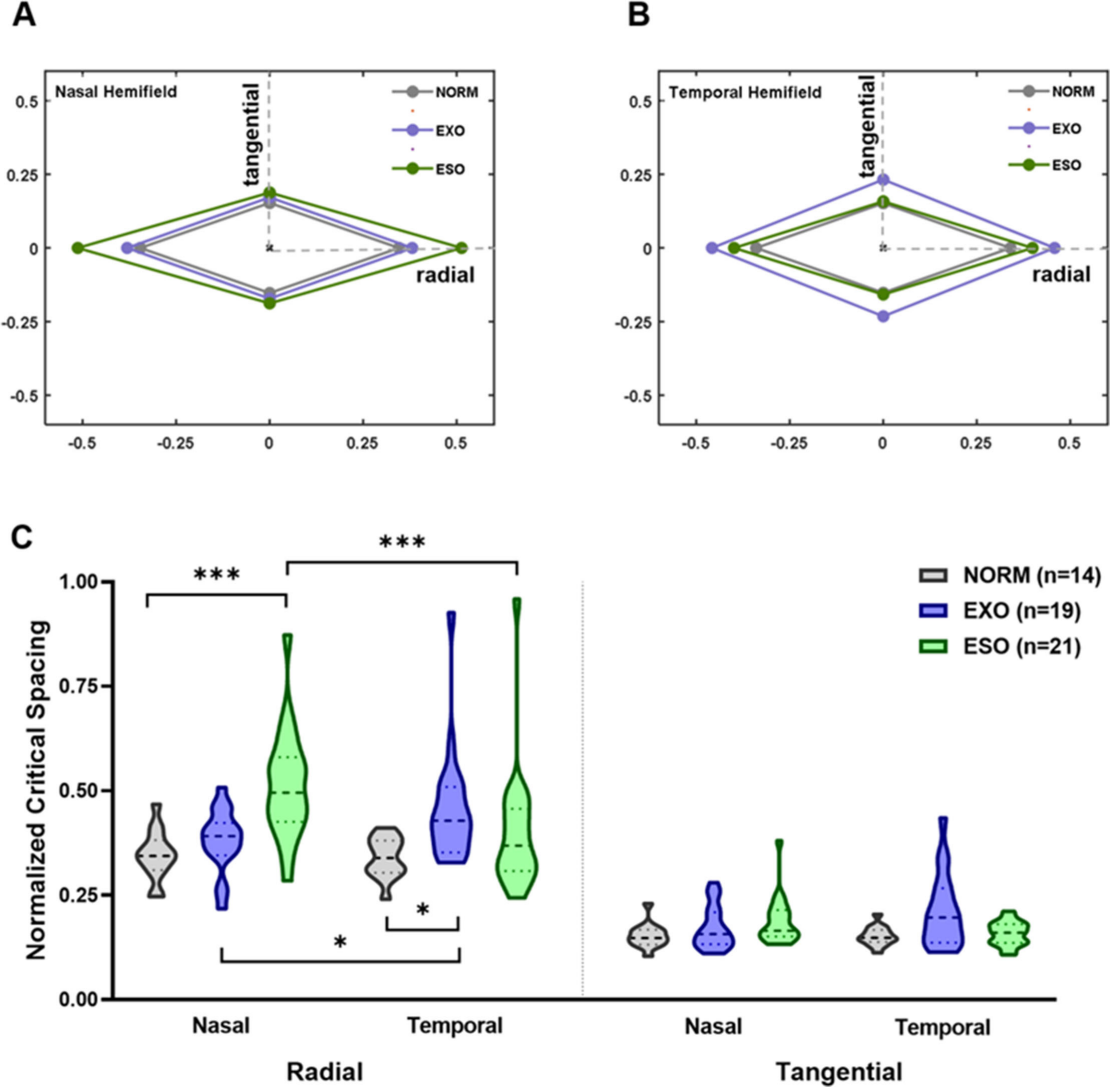
Crowding zones mapped from the average normalized critical spacing in the nasal (**A**) and temporal (**B**) hemifields for the normal, exotropia and esotropia groups. For each hemifield, the size of crowding zone was shown along radial and tangential axes. (**C**) Violin plots of the normalized critical spacing across different hemifields and axes in the normal, exotropia, and esotropia groups. Higher values on the y-axis signify stronger crowding effects. Statistically significant difference: *P < 0.05; ***P < 0.001.

Figure 3 shows the average normalized critical spacing across different hemifields and axes in the normal (NORM), exotropia (EXO), and esotropia (ESO) groups. The average normalized critical spacing of normal controls was approximately consistent with Bouma's law and other estimated values in the literature^12^^,^^34^^,^^36^ in both the nasal hemifield (radial axis = 0.35 ± 0.06, mean ± SE; and tangential axis = 0.15 ± 0.03 × eccentricity) and temporal hemifield (radial axis = 0.34 ± 0.05; and tangential axis = 0.15 ± 0.02 × eccentricity). For the exotropia group, the average normalized critical spacing in the nasal hemifield (radial axis = 0.38 ± 0.07; and tangential axis = 0.17 ± 0.05 × eccentricity) was smaller than that in the temporal hemifield (radial axis = 0.46 ± 0.14; and tangential axis = 0.23 ± 0.16 × eccentricity). For the esotropia group, the average normalized critical spacing in the nasal hemifield (radial axis = 0.51 ± 0.13; and tangential axis = 0.19 ± 0.06 × eccentricity) was larger than that in the temporal hemifield (radial axis = 0.40 ± 0.15; and tangential axis = 0.16 ± 0.03 × eccentricity). An analysis of variance (see Supplementary Table S3 for details) revealed that the normalized critical spacing differed significantly according to axis (F_1,51_ = 365.84; *P* < 0.001), group (F_2,51_ = 3.93; *P* = 0.03), and the interaction of hemifield, axis and group (F_2,51_ = 9.74; *P* < 0.001). Post hoc comparisons revealed that along the radial axis, the normalized critical spacing of patients with exotropia was significantly larger than that of normal controls in the temporal hemifield (EXO versus NORM: t = 3.47, *P* = 0.02), but not in the nasal hemifield (EXO versus NORM: t = 1.03, *P* = 0.99), and the normalized critical spacing in the temporal hemifield was significantly larger than that in the nasal hemifield for patients with exotropia (nasal versus temporal: t = −3.88, *P* = 0.02). In contrast, the normalized critical spacing of patients with esotropia was significantly larger than that of normal controls in the nasal hemifield (ESO versus NORM: t = 5.01, *P* < 0.001) but not in the temporal hemifield (ESO versus NORM: t = 1.77, *P* = 0.83), and the normalized critical spacing in the nasal hemifield was significantly larger than that in the temporal hemifield for patients with esotropia (nasal versus temporal: t = 6.06, *P* < 0.001). No significant difference among the groups was found along the tangential axis (all *P* > 0.05).

In general, we found that visual crowding manifested nasotemporal asymmetry in only the radial axis in patients with strabismus, with patients with exotropia exhibiting stronger peripheral visual crowding in the temporal hemifield (i.e. the deviating direction) and patients with esotropia exhibiting stronger crowding in the nasal hemifield (i.e. the deviating direction) in both the deviated and fixating eyes, implying visual field- and axis-specific cortical miswiring that situated after binocular integration.

### The Altered Visual Crowding is Related to the Strabismus Pattern

**Figure 4.**
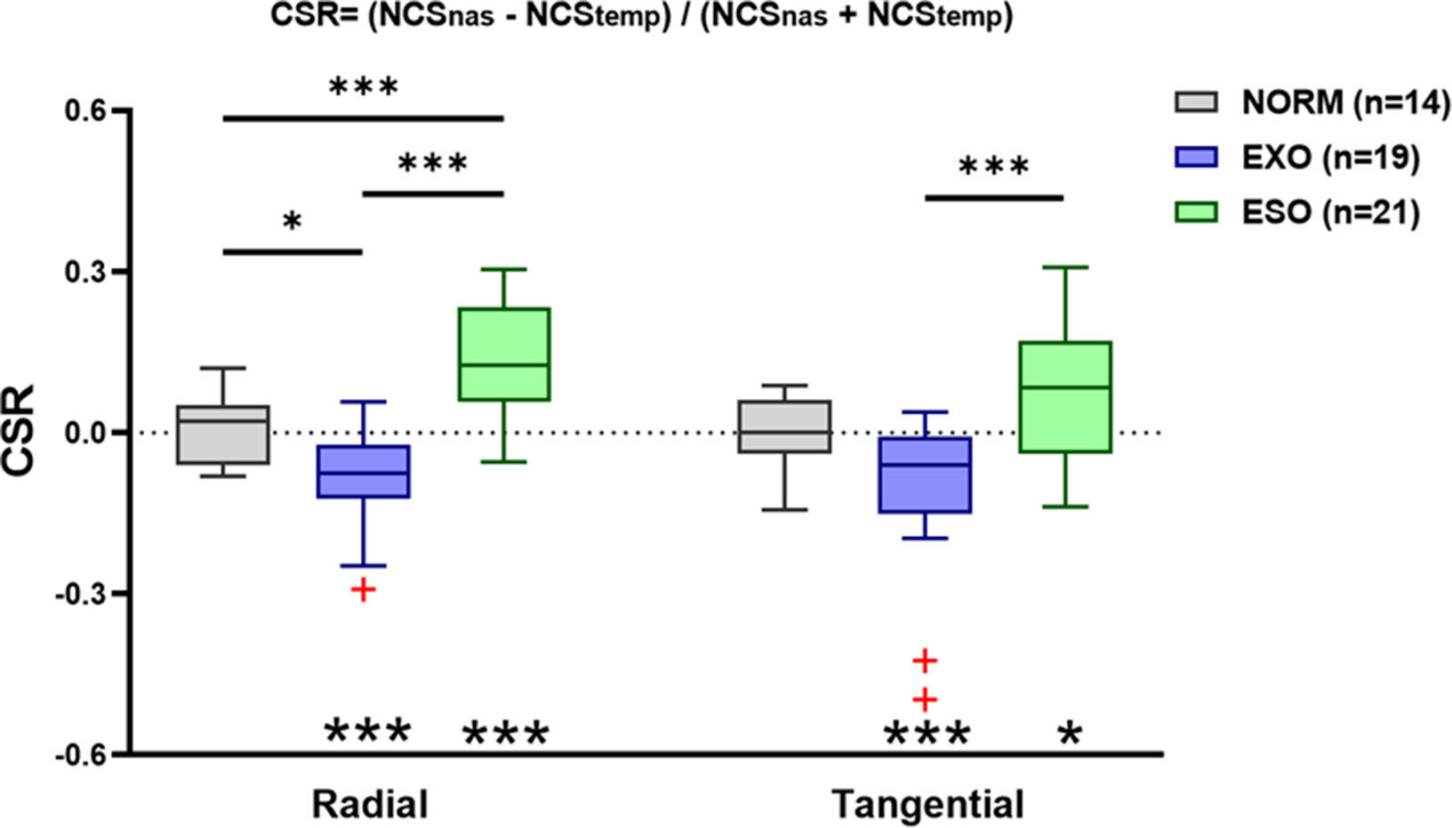
Boxplots of the CSR of normal controls, exotropia and esotropia patients. The *solid line* within each box represents the median. The box represents the interquartile range (IQR) of the data (25th to the 75th percentile). The data points with red crosses represents outliers. The *gray dashed line* indicates CSR of zero. CSR values of <0, 0, and >0 indicates greater crowding effects in the temporal field, symmetrical crowding effects in both visual fields, and greater crowding effects in the nasal field, respectively. Statistically significant difference: *P < 0.05; ***P < 0.001. *Asterisks* below indicate significant differences from zero: *P < 0.05; ***P < 0.001.

To better present the hemifield-specific crowding effects, we calculated the ratio of normalized critical spacing between the nasal and temporal hemifields (the CSR; see Methods) for normal subjects, and patients with exotropia and esotropia (Fig. 4). On average, the CSR was close to zero in normal controls (radial axis = 0.01 ± 0.02, mean ± S.E; and tangential axis = −0.0001 ± 0.02), less than zero in the exotropia group (radial axis = −0.09 ± 0.02; and tangential axis = −0.10 ± 0.03), and larger than zero in the esotropia group (radial axis = 0.13 ± 0.02; and tangential axis = 0.07 ± 0.03). An analysis of variance revealed that the CSR differed significantly among groups along both radial (F_2,51_ = 27.83; *P* < 0.001) and tangential (F_2,51_ = 10.32; *P* < 0.001) axes. Along the radial axis, post hoc comparisons revealed that the CSR of patients with exotropia was smaller than that of normal controls (by 0.09, t = 2.80, *P* < 0.05), and the CSR of patients with esotropia was significantly larger than that of normal controls (by 0.13, t = 3.95, *P* < 0.001) and that of patients with exotropia (by 0.22, t = 7.42, *P* < 0.001). Along the tangential axis, post hoc comparisons revealed a significant difference in the CSR only between esotropia and exotropia patients (by 0.17, t = 4.54, *P* < 0.001).

**Figure 5.**
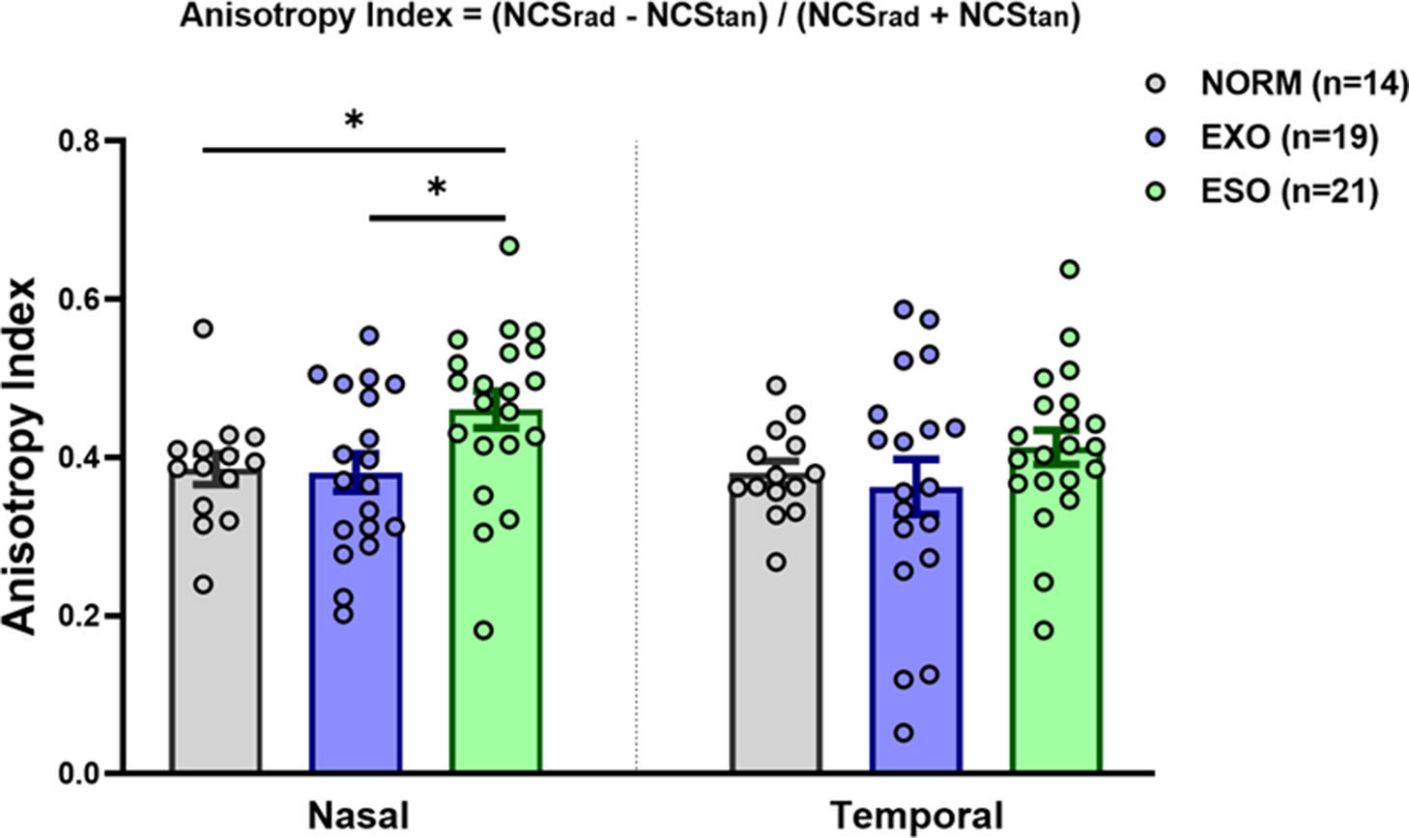
The radial-tangential anisotropy index in the nasal and temporal hemifields of the normal controls, exotropia, and esotropia groups. Error bars are ±1 SEM. Statistically significant difference: *P < 0.05.

We found an increased crowding effects mostly along the radial axis, consistent with the oculomotor deficit in our patients with strabismus (i.e. all had horizontal strabismus). To better visualize the axis-specific crowding effects, the radial-tangential anisotropy index (see Methods) of the 3 groups was calculated, as shown in Figure 5. On average, the radial-tangential anisotropy index of normal controls was 0.39 ± 0.02 (mean ± SE) in the nasal hemifield and 0.38 ± 0.02 in the temporal hemifield. For the exotropia group, the radial-tangential anisotropy index was 0.38 ± 0.02 in the nasal hemifield and 0.36 ± 0.03 in the temporal hemifield; for the esotropia group the corresponding values were 0.46 ± 0.02 and 0.41 ± 0.02. The estimated radial-tangential anisotropy index in normal subjects is largely consistent with other estimated values in the literature.^12^^,^^17^^,^^37^ An analysis of variance revealed that the anisotropy index differed significantly among groups in the nasal (F_2,51_ = 4.05; *P* <0.05) but not temporal (F_2,51_ = 1.01; *P* = 0.37) hemifield. Post hoc comparisons revealed that the anisotropy index of patients with esotropia was greater than that of patients with exotropia (by 0.08, t = 2.57, *P* < 0.05) and normal controls (by 0.08, t = 2.47, *P* < 0.05) in the nasal hemifield. Our results imply that the shape of the crowding zone for patients with esotropia differed from that of normal controls and patients with exotropia; specifically, it was disproportionately enlarged along the radial and tangential axes, indicating altered cortical plasticity.^17^^,^^37^

### Altered Visual Crowding Correlate With Strabismus History

**Figure 6.**
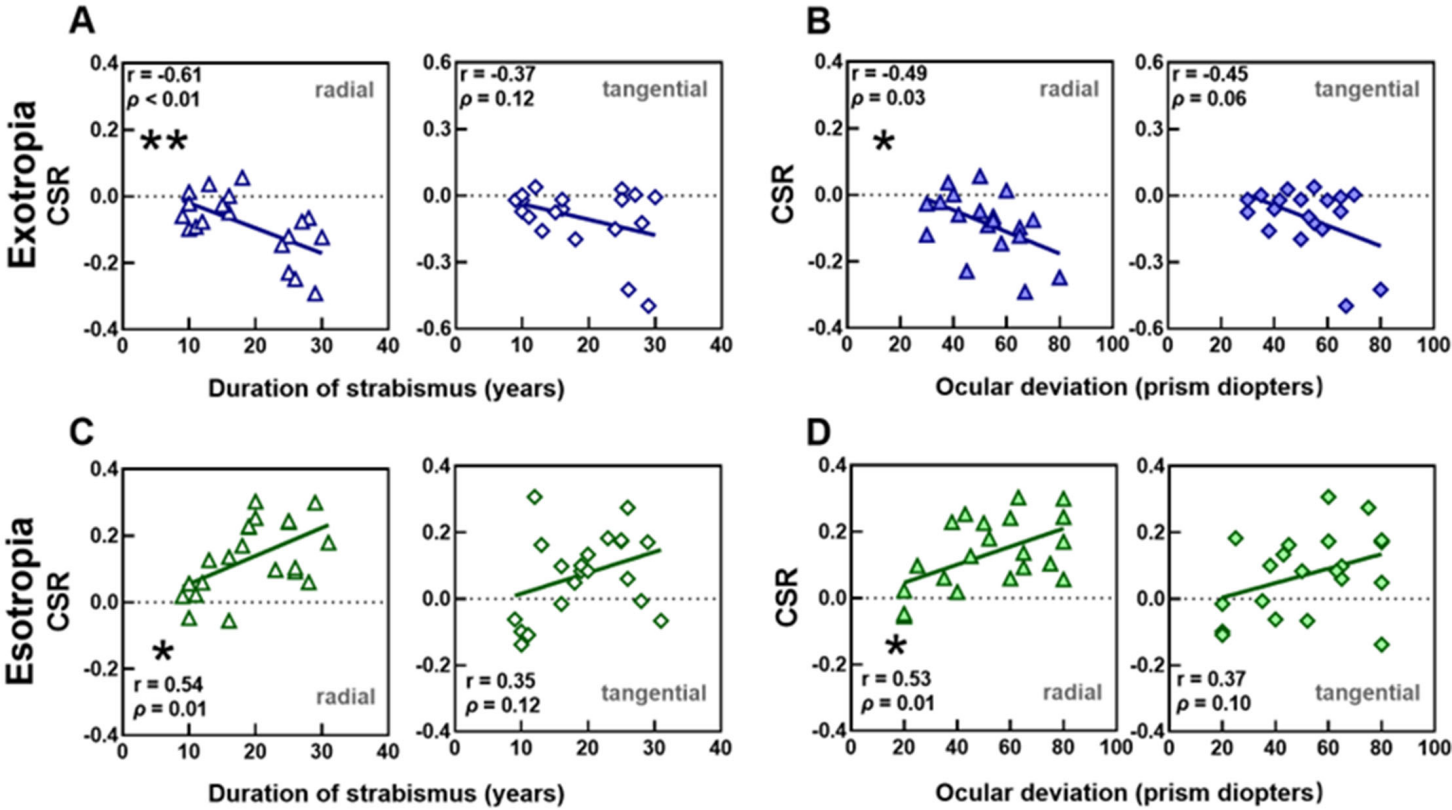
Relationships between the CSR and the duration of strabismus, and between the CSR and the degree of ocular deviation. The abscissa represents the years of duration of strabismus, or the degree of ocular deviation and the ordinate represents the CSR. Each *triangle* or *diamond* represents data of one patient. Asterisks indicate significant correlations: *P < 0.05, **P < 0.01.

We analyzed the relationship between the CSR and patients’ clinical history (e.g. the duration of strabismus and the degree of ocular deviation; Fig. 6). In the exotropia group, the correlation between the CSR and the duration of strabismus was significant along the radial axis (Pearson correlation analysis; r = −0.61, *P* < 0.01), but not along the tangential axis (r = −0.37, *P* = 0.12); additionally, there was a significant correlation between the CSR and the degree of ocular deviation (radial axis: r = −0.49, *P* = 0.03; and tangential axis: r = −0.45, *P* = 0.06). In the esotropia group, similar significant correlations were observed along only the radial axis (correlation between the CSR and the duration of strabismus: r = 0.54, *P* = 0.01; correlation between the CSR and the degree of ocular deviation: r = 0.53, *P* = 0.01), but not along the tangential axis (correlation between the CSR and the duration of strabismus: r = 0.35, *P* = 0.12; correlation between the CSR and the degree of ocular deviation: r = 0.37, *P* = 0.10).

No significant correlation was found between the radial-tangential anisotropy index and patients’ clinical history (see Supplementary Fig. S2; all *P* > 0.10).

## Discussion

In this study, using real-time eye tracking to achieve gaze-contingency, we displayed the target and flankers in specific visual field locations and evaluated visual crowding in patients with horizontal concomitant strabismus without amblyopia. We found that the visual crowding, which imposes a fundamental constraint on object recognition, was greater in the peripheral visual field of patients with long-term axial misalignment and exhibited nasotemporal asymmetry along only the radial axis, with exotropia exhibiting stronger temporal hemifield crowding and esotropia exhibiting stronger nasal hemifield crowding in both the deviated and fixating eyes, which was evidently related to the strabismus pattern. Our results indicate field- and axis-specific cortical miswiring as a result of long-term adaptation to axial misalignment along the horizontal direction.

Our results revealed that in patients with strabismus, critical spacing was larger in the visual field that aligned with the direction of ocular deviation (the temporal visual field of patients with exotropia and the nasal visual field of patients with esotropia). One may argue that all these field-specific deficits may be related to fixational instability or oculomotor bias in strabismus. However, we believe this is unlikely for the following reasons: (1) all of our patients had normal or corrected-to-normal visual acuity (0.00 logMAR or better) and showed good fixation stability during the experiment as monitored by the eye tracker; (2) we performed a 5-point calibration and validation sequence at the beginning of every test to ensure that the validation error was smaller than 1° for all subjects. During the test, the target and flankers were only briefly displayed (250 ms), which also minimized the chance of peeping. (3) We calculated the average fixation duration (total fixation duration/the number of fixations) and found no significant difference in fixation stability among groups, or between the deviated/nondominant eye and fixating/dominant eye in each subgroup. We also analyzed the distribution of fixation points in the nasal and temporal visual fields during the test and generated the fixation map according to the fixation count density. The results showed that the distribution of fixation points in the nasal and temporal hemifields was roughly symmetrical (see Supplementary Fig. S3).

One of the hallmarks of the peripheral visual crowding is radial-tangential anisotropy, in which crowding is 1.5 to 2.5 times stronger for flankers arranged along the radial (meridional) axis than along the tangential (isoeccentric) axis.^10^^,^^12^^,^^34^^,^^35^ Nandy and Tjan attributed this anisotropy to greater spatial extent of crowding for flankers oriented orthogonally to the radial axis compared to those oriented parallel to the radial axis and proposed saccade-confounded image statistics since normal saccadic eye movements are radial with respect to the fovea.^36^ Our results showed significantly greater peripheral visual crowding along only the radial axis in patients with horizontal concomitant strabismus. As the most common type of strabismus, horizontal concomitant strabismus (either exotropia or exotropia) is characterized by horizontal deviation of one eye during fixation and anomalous retinal correspondence between the fovea in the fixating eye and the nonfoveal region in the deviated eye in the horizontal direction.^38^ Because our patients with strabismus essentially did not exhibit vertical deviation in eye position, the crowding effects along the tangential axis was not significantly different from that of the normal controls.

Our results also revealed comparable peripheral visual crowding deficits in both the deviated and fixating eye in patients with exotropia and patients with esotropia. Because all our patients with strabismus had shifted fixation between their two eyes, maintained good central acuity, and did not significantly differ in visual acuity from normal controls, nor between the nasal and temporal hemifields in each subgroup (see Supplementary Fig. S4), our results indicate that crowding has a much more limiting impact than acuity on the capacity of peripheral vision.^18^ Unlike the pattern of monocular crowding deficits in foveal vision, which have been repeatedly demonstrated in both adults and children with strabismic amblyopia,^39^^–^^41^ we revealed binocular increases in peripheral crowding in patients with strabismus without amblyopia in this study. In other words, there might be a monocular crowding deficit in patients with strabismic amblyopia but binocular crowding deficits in patients with strabismus without amblyopia, implying that strabismus with and without amblyopia may be mediated by (at least partially) different mechanisms, despite sharing the symptom of ocular misalignment.

Most of our patients lost stereopsis after long-term axial misalignment. It is not clear if binocular interactions are required for the crowding results. To determine whether there was a difference in the crowding effects depending on the level of binocular function, we compared the normalized critical spacing between patients who retained near stereopsis (only 3 patients with exotropia) and patients who lost stereopsis; we found no significant difference (see Supplementary Table S5). We further compared the normalized critical spacing between patients with normal performance on the Bagolini test (7 patients with exotropia and 8 patients with esotropia) and patients with abnormal performance; again, we found no significant difference (see Supplementary Table S6). More precise quantification of interocular interaction (e.g. suppression) after correction of ocular misalignment, as what we and others have done in previous studies,^42^^,^^43^ can better characterize the relationship between the level of binocular function and the visual crowding in patients with strabismus.

In addition, we found a significant correlation between the critical spacing ratio between the nasal and temporal visual fields (e.g. the CSR) and strabismus duration as well as strabismus angle along the radial axis, suggesting a visual field- and axis-specific adaptation to ocular misalignment. On the one hand, this long-term adaptation seems related only to the deviated direction for any particular patient, because the enhanced crowding occurs regardless of eccentricity (e.g. 5° and 10°). On the other hand, the correction between the CSR and the deviating angle indicates that cortical miswiring is related to the magnitude of deviation. Our results also imply that this adaptation-accompanied cortical miswiring and re-organization may be cumulative and last into late adulthood, indicating the potential of adult brain plasticity and highlighting the importance of ocular correction.

Levi et al. proposed that crowding occurs when an object and its nearest flanker fall within the same region in which integration of visual information occurs.^10^ Whereas in the periphery, these “integration receptive fields” grow larger with the increase of eccentricity, so that close objects are merged into one perception or a vague whole.^19^^,^^44^^–^^46^ In terms of strabismus, some reports have suggested alterations in spontaneous brain activity according to resting-state functional magnetic resonance imaging (fMRI) as well as functional changes in the visual cortex according to blood oxygen level dependent (BOLD)-fMRI in patients with exotropia before and after strabismus surgery.^9^^,^^47^ Animal studies on strabismic cats revealed a squint-induced modification of visual receptive fields in the lateral suprasylvian cortex^48^ and increased receptive field sizes in visual cortical areas A17 and A18.^49^ Thus, it is reasonable that a larger receptive field would lead to more interference between visual stimuli and thus produce stronger crowding effects, which merits further examination in future neuroimaging research into the pathophysiology underlying changes in crowding in patients with strabismus.^50^

The current study has some limitations. First, we focused only on the horizontal visual fields and thus did not globally evaluate the crowding effects in the entire visual field of patients with strabismus. Second, our study was performed in monocular situations with a fixed letter size; however, crowding is considered as a cortical process involving binocular interaction,^11^^,^^12^^,^^51^ more precise quantification of interocular interaction (e.g. suppression) in the mode of binocular presentation after correction of ocular misalignment can better characterize the relationship between the level of binocular function and the crowding in patients with strabismus. To fully understand the impact of crowding in patients with strabismus, future behavioral studies should investigate different stimulus configurations in different fixation states.

## Conclusions

In conclusion, we demonstrated significantly greater peripheral visual crowding with nasotemporal asymmetry along only the radial axis in patients with horizontal strabismus, indicating the existence of hemifield- and axis-specific miswiring of cortical processing in object recognition induced by long-term adaption to ocular misalignment along the horizontal direction.

## Supplementary Material

Supplement 1
