# Supplementary material for: Visual Crowding Reveals Field- and Axis-Specific Cortical Miswiring After Long-Term Axial Misalignment in Strabismic Patients Without Amblyopia
Source: Invest Ophthalmol Vis Sci. 2023 Jan 18;64(1):10. doi: 10.1167/iovs.64.1.10 (PMC9855284; doi:10.1167/iovs.64.1.10)
Supplement: Supplement 1 [file iovs-64-1-10_s001.pdf]

Supplementary Information

Supplementary Table 1. Clinical Characteristics of Strabismic Patients

Patient	Sex/Age	BCVA, DE logMAR	BCVA, FE logMAR	Refraction DE	Refraction FE	Duration of Strabismus (years)	Degree of Strabismus (prism diopters)	Near Stereoacuity (arcsecs)
EXO1	M/12	0.00	0.00	+1.75	+1.25/-0.5*10	10	XT 60	nil
EXO2	F/22	0.00	0.00	PL	-2.75	16	XT 40	nil
EXO3	F/31	0.00	0.00	-3.5/-2.25*170	-3.5/-1.5*180	28	XT 55	nil
EXO4	M/24	0.00	0.00	-3.0/-0.5*100	-1.75/-0.25*125	18	XT 50	nil
EXO5	M/20	0.00	0.00	-2.5/-0.5*175	+0.5/-0.5*160	16	XT 50	nil
EXO6	M/15	-0.10	-0.10	+0.5	PL	10	XT 65	100
EXO7	M/27	0.00	0.00	-0.75	PL	25	XT 45	nil
EXO8	F/37	0.00	0.00	+1.75/-1.5*165	+1.0/-1.25*180	30	XT 65	nil
EXO9	M/13	-0.10	-0.10	PL	-0.5	9	XT 42	40
EXO10	F/27	0.00	0.00	-0.5/-1.0*80	+0.5/-0.5*105	26	XT 80	nil
EXO11	M/21	0.00	0.00	+1.0	+0.25	15	XT 30	nil
EXO12	M/30	0.00	0.00	PL	-1.0	27	XT 70	nil
EXO13	F/21	0.00	0.00	+0.5	+1.0/-1.0*160	13	XT 38	nil
EXO14	M/13	-0.10	-0.10	+1.75	+1.25/-0.5*10	10	XT 35	200
EXO15	M/28	0.00	0.00	-0.75/-0.5*10	-1.25	25	XT 30	nil
EXO16	F/17	0.00	0.00	-2.5/-0.5*180	-1.5/-1.0*170	11	XT 53	nil
EXO17	M/13	0.00	0.00	+1.25/-0.5*10	+1.75	12	XT 55	nil
EXO18	F/36	0.00	0.00	PL	-0.25	29	XT 67	nil
EXO19	M/28	0.00	0.00	-4.5/1.0*165	-5.5/0.5*105	24	XT 58	nil
ESO1	F/28	0.00	0.00	+5.0	+2.0/-0.75*135	28	ET 35	nil
ESO2	M/30	0.00	0.00	-4.5/-1.25*170	-6.5/-0.75*167	29	ET 80	nil
ESO3	M/28	0.00	0.00	+1.5/-1.0*180	+2.0/-0.75*180	20	ET 43	nil
ESO4	F19	0.00	0.00	+0.5	-0.5/-0.5*95	16	ET 65	nil

ESO5	M/26	0.00	0.00	+6.75/-1.25*180	+5.5/-1.25*180	18	ET 80	nil
ESO6	M/27	0.00	0.00	+1.25/-0.5*10	+1.5/-1.5*20	25	ET 60	nil
ESO7	M/30	0.00	0.00	+6.5/-1.0*175	+4.5/-0.75*180	25	ET 80	nil
ESO8	F/32	0.00	0.00	+0.25	/-0.5*155	26	ET 65	nil
ESO9	M/26	0.00	0.00	-2.25/-0.5*170	-2.5/-0.5*20	19	ET 50	nil
ESO10	F/29	0.00	0.00	-4.0/-0.5*170	-4.5	26	ET 75	nil
ESO11	M/25	0.00	0.00	-0.25/-0.5*145	-0.5/-0.5*160	19	ET 38	nil
ESO12	F/13	-0.10	-0.10	+4.5/-1.0*85	+1.25/-0.5*85	9	ET 40	nil
ESO13	M/14	0.00	0.00	+6.5/-1.50*180	+5.5/-1.25*180	12	ET 60	nil
ESO14	F/18	0.00	0.00	+3.75/-2.25*20	+1.00/-0.5*5	13	ET 45	nil
ESO15	F/19	0.00	0.00	-1.5/0.5*95	-2.0	16	ET 20	nil
ESO16	F/28	0.00	0.00	-0.25	PL	23	ET 25	nil
ESO17	F/33	0.00	0.00	+0.5/-1.0*100	-0.5	31	ET 52	nil
ESO18	M/28	0.00	0.00	+1.75/-1.5*90	+3.5/-1.25*15	20	ET 63	nil
ESO19	M/13	0.00	0.00	+3.5/-2.25*180	+5.0/-2.0*3	10	ET 20	nil
ESO20	F/14	-0.10	-0.10	+1.0	-0.5/-0.5*95	10	ET 80	nil
ESO21	F/13	0.00	0.00	+3.5/-0.5*55	+2.50/-0.5*120	11	ET 20	nil

ESO/ET: esotropia; EXO/XT: exotropia; DE: deviated eye; FE: fixating eye; BCVA: best-corrected visual acuity.

Supplementary Table 2. Results of an analysis of variance (ANOVA) performed to the normalized critical spacing

Within Subjects Effects

Cases	Sum of Squares	df	Mean Square	F	p
eye	2.869e -5	1	2.869e -5	0.001	0.975
eye ✻ group	0.064	2	0.032	1.142	0.329
Residuals	1.114	40	0.028		
hemifield	0.045	1	0.045	5.734	0.021
hemifield ✻ group	0.517	2	0.258	32.998	< .001
Residuals	0.313	40	0.008		
axis	8.935	1	8.935	629.263	< .001
axis ✻ group	0.218	2	0.109	7.660	0.002
Residuals	0.568	40	0.014		
eccentricity	0.015	1	0.015	1.247	0.271
eccentricity ✻ group	0.027	2	0.014	1.131	0.333
Residuals	0.485	40	0.012		
eye ✻ hemifield	0.014	1	0.014	0.922	0.343
eye ✻ hemifield ✻ group	0.016	2	0.008	0.536	0.589
Residuals	0.607	40	0.015		
eye ✻ axis	9.545e -4	1	9.545e -4	0.146	0.704
eye ✻ axis ✻ group	0.004	2	0.002	0.310	0.735
Residuals	0.262	40	0.007		
hemifield ✻ axis	0.028	1	0.028	4.857	0.033
hemifield ✻ axis ✻ group	0.080	2	0.040	7.014	0.002
Residuals	0.228	40	0.006		
eye ✻ eccentricity	7.112e -4	1	7.112e -4	0.128	0.723
eye ✻ eccentricity ✻ group	0.022	2	0.011	1.958	0.154
Residuals	0.223	40	0.006		
hemifield ✻ eccentricity	0.002	1	0.002	0.284	0.597
hemifield ✻ eccentricity ✻ group	0.005	2	0.002	0.378	0.688
Residuals	0.244	40	0.006		
axis ✻ eccentricity	0.031	1	0.031	4.011	0.052
axis ✻ eccentricity ✻ group	0.023	2	0.011	1.459	0.245
Residuals	0.312	40	0.008		
eye ✻ hemifield ✻ axis	4.380e -5	1	4.380e -5	0.007	0.933
eye ✻ hemifield ✻ axis ✻ group	0.011	2	0.005	0.881	0.422
Residuals	0.246	40	0.006		
eye ✻ hemifield ✻ eccentricity	0.066	1	0.066	10.484	0.002
eye ✻ hemifield ✻ eccentricity ✻ group	0.003	2	0.002	0.263	0.770
Residuals	0.252	40	0.006		
eye ✻ axis ✻ eccentricity	1.903e -4	1	1.903e -4	0.046	0.831
eye ✻ axis ✻ eccentricity ✻ group	0.002	2	9.336e -4	0.226	0.799
Residuals	0.165	40	0.004		

Within Subjects Effects

Cases	Sum of Squares	df	Mean Square	F	p
hemifield ✻ axis ✻ eccentricity	8.803e -4	1	8.803e -4	0.422	0.520
hemifield ✻ axis ✻ eccentricity ✻ group	0.004	2	0.002	0.902	0.414
Residuals	0.083	40	0.002		
eye ✻ hemifield ✻ axis ✻ eccentricity	0.009	1	0.009	1.825	0.184
eye ✻ hemifield ✻ axis ✻ eccentricity ✻ group	0.003	2	0.002	0.348	0.708
Residuals	0.186	40	0.005		

Note. Type III Sum of Squares

Between Subjects Effects

Cases	Sum of Squares	df	Mean Square	F	p
group	0.515	2	0.258	9.328	< .001
Residuals	1.105	40	0.028		

Note. Type III Sum of Squares

Supplementary Table 3. Results of an analysis of variance (ANOVA) performed to the mean normalized critical spacing across eyes and eccentricities

Within Subjects Effects

Cases	Sum of Squares	df	Mean Square	F	p
hemifield	2.795e -4	1	2.795e -4	0.046	0.830
hemifield ✻ group	0.196	2	0.098	16.310	< .001
Residuals	0.307	51	0.006		
axis	2.777	1	2.777	365.843	< .001
axis ✻ group	0.082	2	0.041	5.406	0.007
Residuals	0.387	51	0.008		
hemifield ✻ axis	0.008	1	0.008	5.401	0.024
hemifield ✻ axis ✻ group	0.029	2	0.014	9.744	< .001
Residuals	0.075	51	0.001		

Note. Type III Sum of Squares

Between Subjects Effects

Cases	Sum of Squares	df	Mean Square	F	p
group	0.176	2	0.088	3.926	0.026
Residuals	1.142	51	0.022		

Note. Type III Sum of Squares

Post Hoc Comparisons - group ✻ hemifield ✻ axis

		Mean Difference	SE	t	p_{bonf}	
ESO, nasal, radial	EXO, nasal, radial	0.132	0.031	4.312	0.002	**
	NORM, nasal, radial	0.167	0.033	5.006	< .001	***
	ESO, temporal, radial	0.114	0.019	6.062	< .001	***
	EXO, temporal, radial	0.055	0.031	1.802	1.000	
	NORM, temporal, radial	0.173	0.033	5.193	< .001	***
	ESO, nasal, tangential	0.326	0.021	15.674	< .001	***
	EXO, nasal, tangential	0.341	0.031	11.128	< .001	***
	NORM, nasal, tangential	0.360	0.033	10.784	< .001	***
	ESO, temporal, tangential	0.355	0.025	13.934	< .001	***
	EXO, temporal, tangential	0.281	0.031	9.170	< .001	***
	NORM, temporal, tangential	0.361	0.033	10.810	< .001	***
EXO, nasal, radial	NORM, nasal, radial	0.035	0.034	1.027	1.000	
	ESO, temporal, radial	-0.018	0.031	-0.578	1.000	

Post Hoc Comparisons - group ✻ hemifield ✻ axis

		Mean Difference	SE	t	p_{bonf}	
	EXO, temporal, radial	-0.077	0.020	-3.875	0.015	*
	NORM, temporal, radial	0.041	0.034	1.211	1.000	
	ESO, nasal, tangential	0.193	0.031	6.310	< .001	***
	EXO, nasal, tangential	0.209	0.022	9.567	< .001	***
	NORM, nasal, tangential	0.228	0.034	6.687	< .001	***
	ESO, temporal, tangential	0.223	0.031	7.262	< .001	***
	EXO, temporal, tangential	0.149	0.027	5.563	< .001	***
	NORM, temporal, tangential	0.229	0.034	6.713	< .001	***
NORM, nasal, radial	ESO, temporal, radial	-0.053	0.033	-1.579	1.000	
	EXO, temporal, radial	-0.112	0.034	-3.283	0.089	
	NORM, temporal, radial	0.006	0.023	0.271	1.000	
	ESO, nasal, tangential	0.158	0.033	4.742	< .001	***
	EXO, nasal, tangential	0.174	0.034	5.100	< .001	***
	NORM, nasal, tangential	0.193	0.025	7.586	< .001	***
	ESO, temporal, tangential	0.188	0.033	5.615	< .001	***
	EXO, temporal, tangential	0.114	0.034	3.340	0.074	
	NORM, temporal, tangential	0.194	0.031	6.217	< .001	***
ESO, temporal, radial	EXO, temporal, radial	-0.059	0.031	-1.932	1.000	
	NORM, temporal, radial	0.059	0.033	1.766	1.000	
	ESO, nasal, tangential	0.211	0.025	8.292	< .001	***
	EXO, nasal, tangential	0.227	0.031	7.394	< .001	***
	NORM, nasal, tangential	0.246	0.033	7.357	< .001	***
	ESO, temporal, tangential	0.240	0.021	11.568	< .001	***
	EXO, temporal, tangential	0.167	0.031	5.436	< .001	***
	NORM, temporal, tangential	0.247	0.033	7.383	< .001	***
EXO, temporal, radial	NORM, temporal, radial	0.118	0.034	3.467	0.048	*
	ESO, nasal, tangential	0.270	0.031	8.820	< .001	***
	EXO, nasal, tangential	0.286	0.027	10.679	< .001	***
	NORM, nasal, tangential	0.305	0.034	8.943	< .001	***
	ESO, temporal, tangential	0.299	0.031	9.772	< .001	***
	EXO, temporal, tangential	0.226	0.022	10.341	< .001	***
	NORM, temporal, tangential	0.306	0.034	8.969	< .001	***
NORM, temporal, radial	ESO, nasal, tangential	0.152	0.033	4.554	< .001	***
	EXO, nasal, tangential	0.168	0.034	4.916	< .001	***
	NORM, nasal, tangential	0.187	0.031	5.988	< .001	***
	ESO, temporal, tangential	0.181	0.033	5.428	< .001	***
	EXO, temporal, tangential	0.108	0.034	3.156	0.134	
	NORM, temporal, tangential	0.188	0.025	7.374	< .001	***

Post Hoc Comparisons - group ✻ hemifield ✻ axis

		Mean Difference	SE	t	p_{bonf}
ESO, nasal, tangential	EXO, nasal, tangential	0.016	0.031	0.506	1.000
	NORM, nasal, tangential	0.035	0.033	1.036	1.000
	ESO, temporal, tangential	0.029	0.019	1.546	1.000
	EXO, temporal, tangential	-0.044	0.031	-1.452	1.000
	NORM, temporal, tangential	0.035	0.033	1.063	1.000
EXO, nasal, tangential	NORM, nasal, tangential	0.019	0.034	0.560	1.000
	ESO, temporal, tangential	0.014	0.031	0.446	1.000
	EXO, temporal, tangential	-0.060	0.020	-3.023	0.226
	NORM, temporal, tangential	0.020	0.034	0.586	1.000
NORM, nasal, tangential	ESO, temporal, tangential	-0.005	0.033	-0.163	1.000
	EXO, temporal, tangential	-0.079	0.034	-2.320	1.000
	NORM, temporal, tangential	8.750e -4	0.023	0.038	1.000
ESO, temporal, tangential	EXO, temporal, tangential	-0.074	0.031	-2.404	1.000
	NORM, temporal, tangential	0.006	0.033	0.189	1.000
EXO, temporal, tangential	NORM, temporal, tangential	0.080	0.034	2.346	1.000

* p < .05, ** p < .01, *** p < .001

Note. P-value adjusted for comparing a family of 66

Supplementary Table 4. Results of ANOVA performed to the mean normalized critical spacing across eyes and eccentricities for patients who completed the tests with both eyes.

Within Subjects Effects

Cases	Sum of Squares	df	Mean Square	F	p
hemifield	0.008	1	0.008	4.062	0.051
hemifield ✻ group	0.142	2	0.071	36.375	< .001
Residuals	0.078	40	0.002		
axis	2.184	1	2.184	671.128	< .001
axis ✻ group	0.057	2	0.028	8.725	< .001
Residuals	0.130	40	0.003		
hemifield ✻ axis	0.004	1	0.004	2.796	0.102
hemifield ✻ axis ✻ group	0.025	2	0.012	7.843	0.001
Residuals	0.063	40	0.002		

Note. Type III Sum of Squares

Between Subjects Effects

Cases	Sum of Squares	df	Mean Square	F	p
group	0.129	2	0.064	9.249	< .001
Residuals	0.279	40	0.007		

Note. Type III Sum of Squares

Post Hoc Comparisons - group ✻ hemifield ✻ axis

		Mean Difference	SE	t	p_{bonf}	
ESO, nasal, radial	EXO, nasal, radial	0.160	0.022	7.324	< .001	***
	NORM, nasal, radial	0.170	0.021	7.903	< .001	***
	ESO, temporal, radial	0.130	0.015	8.752	< .001	***
	EXO, temporal, radial	0.095	0.022	4.358	0.002	**
	NORM, temporal, radial	0.176	0.021	8.195	< .001	***
	ESO, nasal, tangential	0.316	0.017	18.208	< .001	***
	EXO, nasal, tangential	0.360	0.022	16.443	< .001	***
	NORM, nasal, tangential	0.363	0.021	16.893	< .001	***
	ESO, temporal, tangential	0.362	0.018	20.073	< .001	***
	EXO, temporal, tangential	0.324	0.022	14.787	< .001	***

Post Hoc Comparisons - group ✻ hemifield ✻ axis

		Mean Difference	SE	t	p bonf	
	NORM, temporal, tangential	0.363	0.021	16.934	< .001	***
EXO, nasal, radial	NORM, nasal, radial	0.009	0.023	0.409	1.000	
	ESO, temporal, radial	-0.030	0.022	-1.380	1.000	
	EXO, temporal, radial	-0.065	0.016	-3.937	0.012	*
	NORM, temporal, radial	0.016	0.023	0.687	1.000	
	ESO, nasal, tangential	0.156	0.022	7.129	< .001	***
	EXO, nasal, tangential	0.200	0.019	10.356	< .001	***
	NORM, nasal, tangential	0.202	0.023	8.951	< .001	***
	ESO, temporal, tangential	0.202	0.022	9.221	< .001	***
	EXO, temporal, tangential	0.163	0.020	8.162	< .001	***
	NORM, temporal, tangential	0.203	0.023	8.990	< .001	***
NORM, nasal, radial	ESO, temporal, radial	-0.039	0.021	-1.839	1.000	
	EXO, temporal, radial	-0.074	0.023	-3.284	0.089	
	NORM, temporal, radial	0.006	0.016	0.394	1.000	
	ESO, nasal, tangential	0.147	0.021	6.843	< .001	***
	EXO, nasal, tangential	0.190	0.023	8.432	< .001	***
	NORM, nasal, tangential	0.193	0.019	10.384	< .001	***
	ESO, temporal, tangential	0.193	0.021	8.978	< .001	***
	EXO, temporal, tangential	0.154	0.023	6.826	< .001	***
	NORM, temporal, tangential	0.194	0.019	10.045	< .001	***
ESO, temporal, radial	EXO, temporal, radial	-0.035	0.022	-1.585	1.000	
	NORM, temporal, radial	0.046	0.021	2.131	1.000	
	ESO, nasal, tangential	0.186	0.018	10.323	< .001	***
	EXO, nasal, tangential	0.230	0.022	10.500	< .001	***
	NORM, nasal, tangential	0.232	0.021	10.829	< .001	***
	ESO, temporal, tangential	0.232	0.017	13.357	< .001	***
	EXO, temporal, tangential	0.194	0.022	8.843	< .001	***
	NORM, temporal, tangential	0.233	0.021	10.870	< .001	***
EXO, temporal, radial	NORM, temporal, radial	0.080	0.023	3.562	0.035	*
	ESO, nasal, tangential	0.221	0.022	10.094	< .001	***
	EXO, nasal, tangential	0.265	0.020	13.216	< .001	***
	NORM, nasal, tangential	0.267	0.023	11.826	< .001	***
	ESO, temporal, tangential	0.267	0.022	12.187	< .001	***
	EXO, temporal, tangential	0.228	0.019	11.843	< .001	***
	NORM, temporal, tangential	0.268	0.023	11.865	< .001	***

Post Hoc Comparisons - group ✻ hemifield ✻ axis

		Mean Difference	SE	t	p_{bonf}
NORM, temporal, radial	ESO, nasal, tangential	0.141	0.021	6.551	< .001 ***
	EXO, nasal, tangential	0.184	0.023	8.154	< .001 ***
	NORM, nasal, tangential	0.187	0.019	9.675	< .001 ***
	ESO, temporal, tangential	0.186	0.021	8.686	< .001 ***
	EXO, temporal, tangential	0.148	0.023	6.548	< .001 ***
	NORM, temporal, tangential	0.188	0.019	10.094	< .001 ***
ESO, nasal, tangential	EXO, nasal, tangential	0.044	0.022	1.991	1.000
	NORM, nasal, tangential	0.046	0.021	2.147	1.000
	ESO, temporal, tangential	0.046	0.015	3.082	0.187
	EXO, temporal, tangential	0.007	0.022	0.334	1.000
	NORM, temporal, tangential	0.047	0.021	2.188	1.000
EXO, nasal, tangential	NORM, nasal, tangential	0.002	0.023	0.110	1.000
	ESO, temporal, tangential	0.002	0.022	0.102	1.000
	EXO, temporal, tangential	-0.036	0.016	-2.199	1.000
	NORM, temporal, tangential	0.003	0.023	0.149	1.000
NORM, nasal, tangential	ESO, temporal, tangential	-2.589e -4	0.021	-0.012	1.000
	EXO, temporal, tangential	-0.039	0.023	-1.716	1.000
	NORM, temporal, tangential	8.750e -4	0.016	0.055	1.000
ESO, temporal, tangential	EXO, temporal, tangential	-0.039	0.022	-1.758	1.000
	NORM, temporal, tangential	0.001	0.021	0.053	1.000
EXO, temporal, tangential	NORM, temporal, tangential	0.040	0.023	1.755	1.000

* $p < .05$, ** $p < .01$, *** $p < .001$

Note. P-value adjusted for comparing a family of 66

Supplementary Table 5. Normalized critical spacing (mean ± SD) for patients with and without stereopsis

Exotropia	Nasal		Temporal	
	radial	tangential	radial	tangential
With stereopsis (n=3)	0.3995 ± 0.044	0.183 ± 0.057	0.447 ± 0.060	0.195 ± 0.064
Without stereopsis （n=16）	0.378 ± 0.079	0.170 ± 0.052	0.459 ± 0.147	0.209 ± 0.074

Supplementary Table 6. Normalized critical spacing (mean ± SD) for patients with normal and abnormal Bagolini test results

Group	Bagolini test	Nasal		Temporal	
		radial	tangential	radial	tangential
Exotropia	Normal(n=7)	0.390 ± 0.067	0.170 ± 0.058	0.460 ± 0.093	0.196 ± 0.083
	Abnormal(n=12)	0.375 ± 0.080	0.173 ± 0.049	0.457 ± 0.159	0.219 ± 0.091
Esotropia	Normal(n=8)	0.506 ± 0.083	0.185 ± 0.035	0.420 ± 0.115	0.164 ± 0.030
	Abnormal(n=13)	0.517 ± 0.072	0.189 ± 0.064	0.385 ± 0.078	0.155 ± 0.026

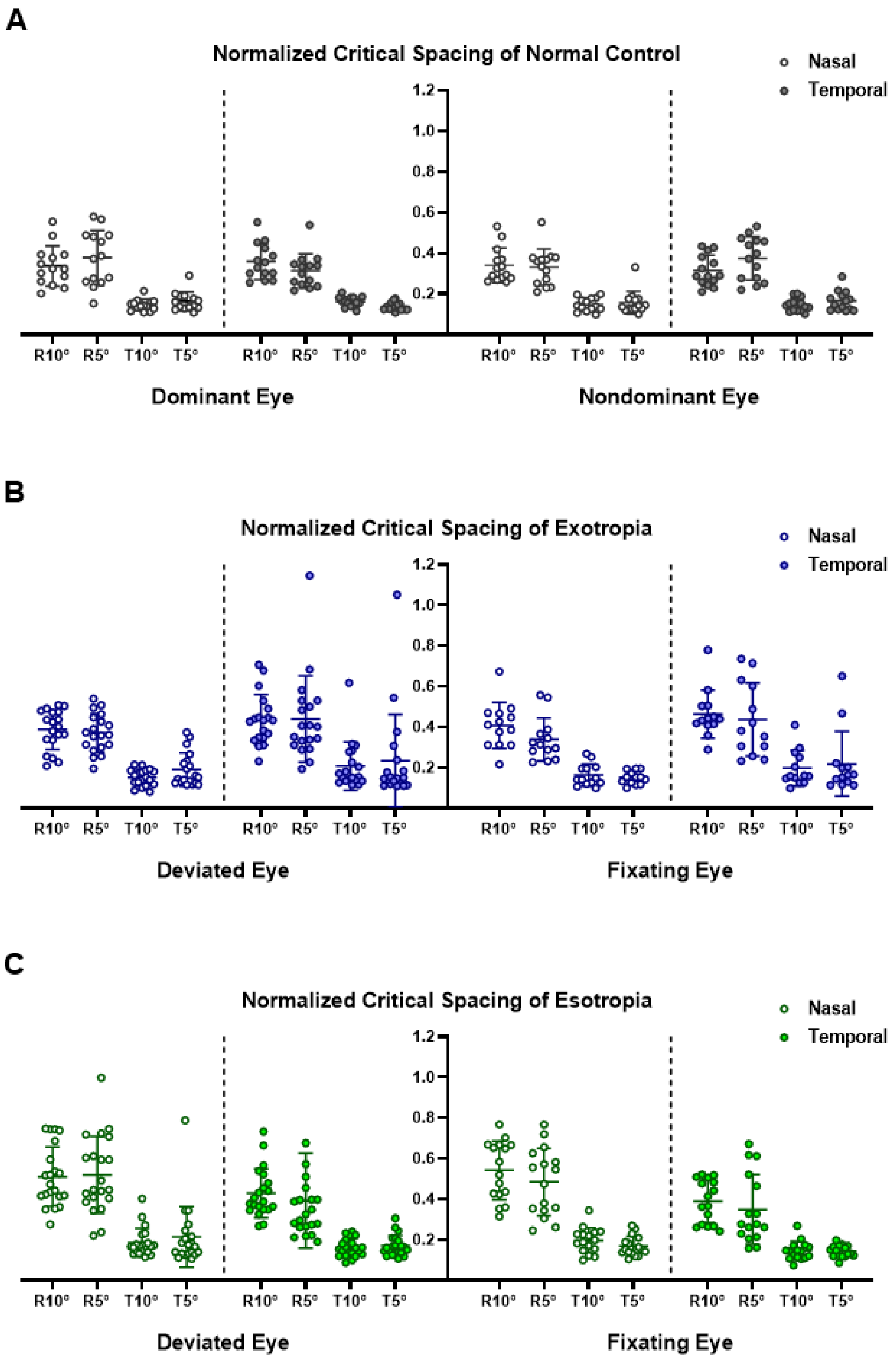

FIGURE S1. The normalized critical spacing in different groups (A: normal controls, B: exotropia group, C: esotropia group), hemifields, eyes, eccentricities and axes. R10° represents along the radial axis and at the eccentricity of 10°; R5° represents along the radial axis and at the

eccentricity of 5°; T10° represents along the tangential axis and at the eccentricity of 10°; T5° represents along the tangential axis and at the eccentricity of 5°。Each circle represents data of one participant.

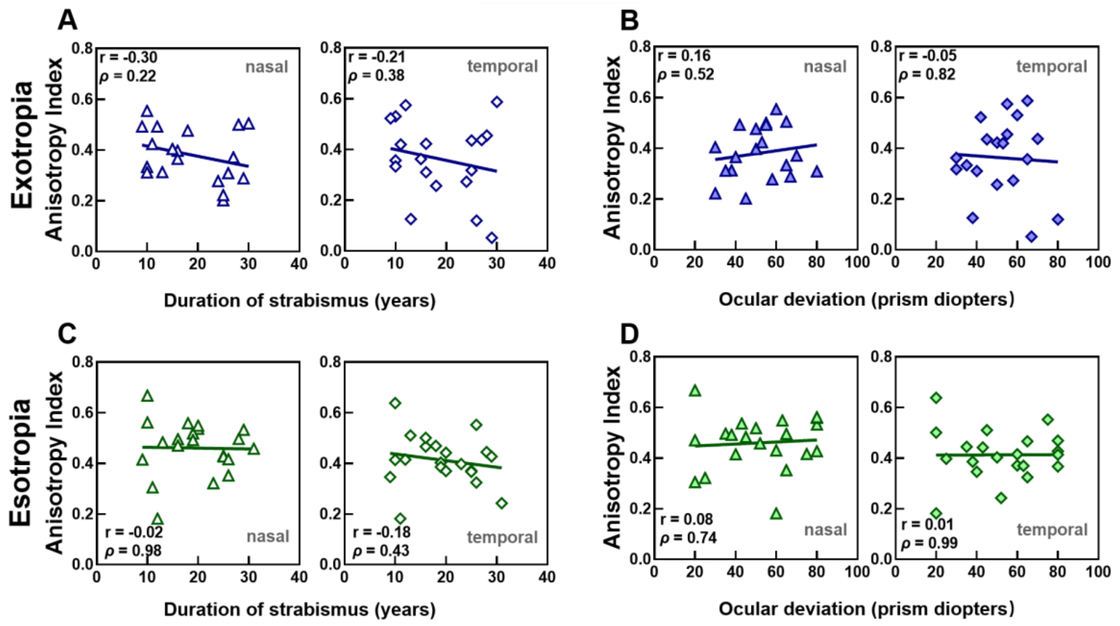

FIGURE S2. Relationships between the anisotropy index and the duration of strabismus, and between the anisotropy index and the degree of ocular deviation. The abscissa represents the years of duration of strabismus, or the degrees of ocular deviation and the ordinate represents the anisotropy index. Each triangle or diamond represents data of one patient.

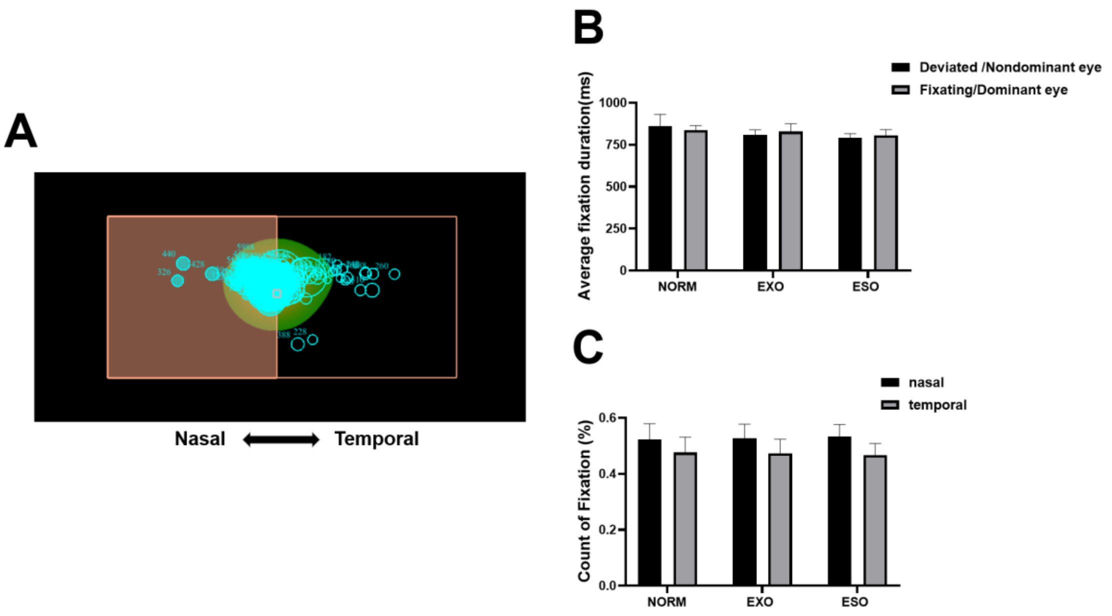

FIGURE S3. (A) A spatial overlay of fixation points during a crowding test from a representative exotropia patient. Each blue circle represents a fixation point and the white square box represents the first fixation point at the beginning of the test. We divided the distribution view into the nasal and temporal visual field according to the position of the fixed point in the center of the screen and then counted the number of fixation points on both sides separately. (B) Comparisons of the average fixation duration (total fixation duration/the number of fixations) across eyes in different groups. Higher values on the y-axis signify greater fixation stability. Error bars are ±1 SEM. (C) Comparisons of the proportion of fixation points between the nasal and temporal hemifields in different groups. Higher values on the y-axis signify greater fixation tendency. Error bars are ±1 SEM.

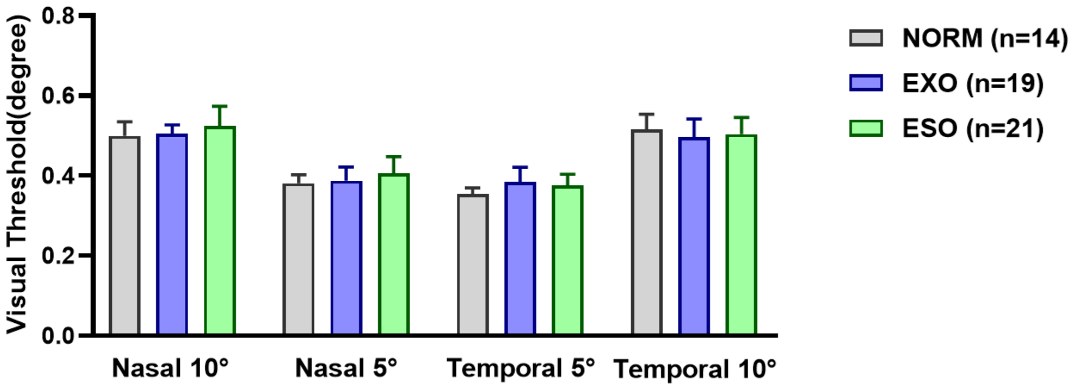

FIGURE S4. The results of visual acuity test in different visual field locations of normal controls, exotropia and esotropia groups. Y-axis represents the minimum visible letter size. Error bars are ±1 SEM.

To further clarify that the crowding effect is equivalently elevated in both eyes for the strabismic group, we conducted independent samples t-tests of the temporal VS nasal difference in the deviated and fixating eyes separately for the exotropia and esotropia group. The result was consistent with the post-hoc comparisons in the ANOVA analysis.

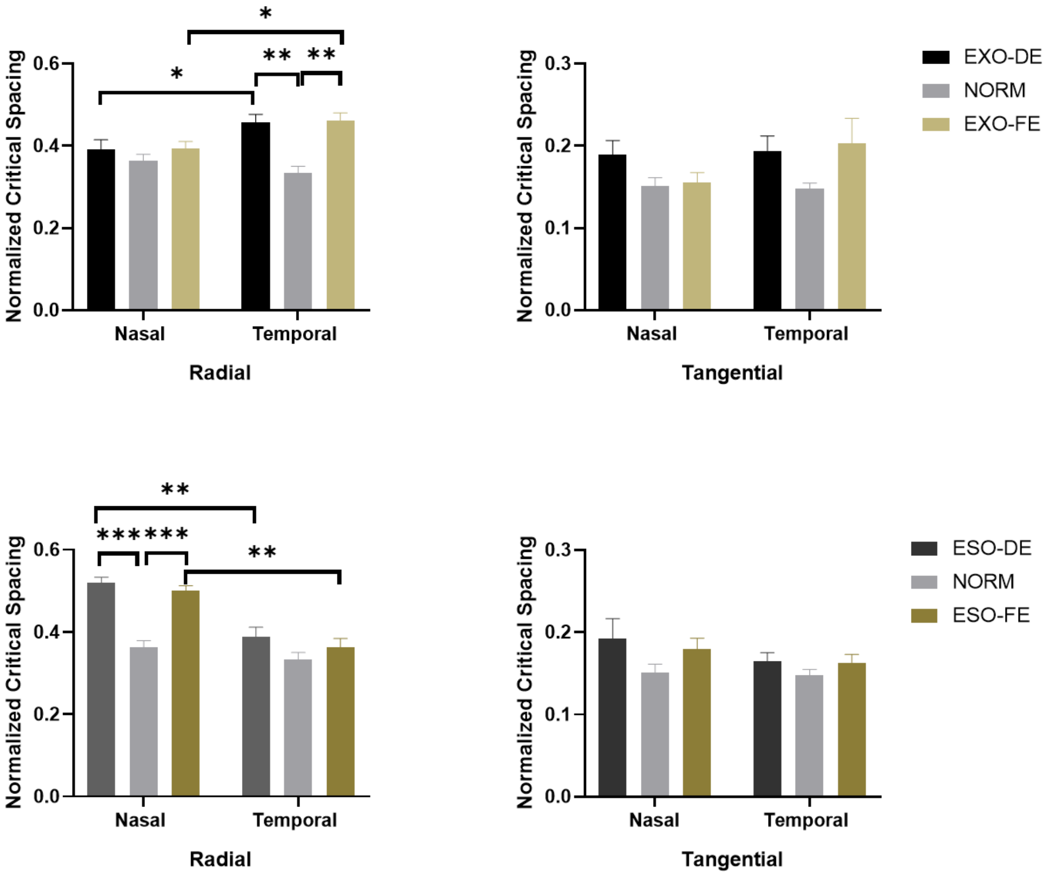

Figure S5. Results of independent t-tests of the normalized critical spacing across different hemifields and axes for strabismic patients at the deviated and fixating eye. Higher values on the y-axis signify stronger crowding effect. Statistically significance: * P < 0.05, **P < 0.01, *** P < 0.001.
